# *Coriolus versicolor* biomass increases dendritic arborization of newly-generated neurons in mouse hippocampal dentate gyrus

**DOI:** 10.18632/oncotarget.25978

**Published:** 2018-08-31

**Authors:** Elisabete Ferreiro, Inês R. Pita, Sandra I. Mota, Jorge Valero, Nuno R. Ferreira, Tito Fernandes, Vittorio Calabrese, Carlos A. Fontes-Ribeiro, Frederico C. Pereira, Ana Cristina Rego

**Affiliations:** ^1^ CNC-Center for Neuroscience and Cell Biology, University of Coimbra, Coimbra, Portugal; ^2^ III-Institute for Interdisciplinary Research (IIIUC), University of Coimbra, Coimbra, Portugal; ^3^ Institute of Pharmacology and Experimental Therapeutics/IBILI, Faculty of Medicine, University of Coimbra, Coimbra, Portugal; ^4^ CNC.IBILI–University of Coimbra, Coimbra, Portugal; ^5^ Achucarro Basque Center for Neuroscience, Science Park of the University of the Basque Country (UPV/EHU), Leioa, Spain; ^6^ Ikerbasque Basque Foundation for Science, Bilbao, Bizkaia, Spain; ^7^ Faculty of Pharmacy, University of Coimbra, Coimbra, Portugal; ^8^ Ministry of Education, Maputo, Mozambique; ^9^ Faculty of Veterinary Medicine, Lisbon University, Lisbon, Portugal; ^10^ Department of Biomedical and Biotechnological Sciences, School of Medicine, University of Catania, Catania, Italy; ^11^ Institute of Biochemistry, Faculty of Medicine, University of Coimbra, Coimbra, Portugal

**Keywords:** coriolus versicolor, cognitive reserve, hippocampus, neurogenesis, immature neuron complexity

## Abstract

Brain cognitive reserve refers to the ability of the brain to manage different challenges that arise throughout life, making it resilient to neuropathology. Hippocampal adult neurogenesis has been considered to be a relevant contributor for brain cognitive reserve and brain plasticity. *Coriolus versicolor* (CV), a common healthful mushroom, has been receiving increasing attention by its antitumoral, anti-inflammatory, antioxidant, antibacterial, and immunomodulatory properties, including in the hippocampus. Herein, we evaluated whether CV biomass oral administration for 2.5 months enhances hippocampal neurogenic reserve under normal/physiological conditions, by quantifying hippocampal dentate gyrus (DG) granular cell layer (GCL) and subgranular zone (SGZ) volumes, proliferation, number and dendritic complexity features of hippocampal newly-generated neurons. We also analyzed β-catenin levels in DG newly-generated immature neurons, because it plays a major role in neurogenesis. Although no differences were observed in the volume of GCL and SGZ layers, in proliferation and in the number of newly-generated neurons of controls and CV-administered mice, we found that CV administration promotes a significant increase in dendritic length and branching and total dendritic volume of immature neurons, suggesting a positive effect of oral CV administration in the hippocampal neurogenic reserve. We also observed that β-catenin levels are increased both in the nucleus and cytoplasm of DG immature neurons, suggesting that Wnt/β-catenin signalling may play an important role in the CV positive effect on the differentiation of these cells. These data unveil a so far unexplored neurogenic potential of CV supplementation, which emerges as a possible preventive strategy for different neurological conditions.

## INTRODUCTION

The brain must cope with different challenges throughout life. The capacity of the brain to respond to these insults may define the difference between undergoing healthy aging or developing a neurological disease. Brain cognitive reserve reflects the brain capacity to preserve normal function [1]. Several factors modulate the cognitive reserve, namely nutrition, stress, exercise and environmental enrichment. Albeit the difficulty to evaluate the cognitive reserve status, different parameters have been used to assess brain function potential, such as brain volume, neuronal number and dendritic complexity [1]. Particularly, hippocampal adult neurogenesis has been pointed out as a putative contributor to the cognitive reserve [2]. The production of adult hippocampal neurons requires the continuous division of neural stem/progenitor cells, differentiation of newly-generated granule cells and their integration into the pre-existing circuits under specified extracellular conditions [3]. This integration into the hippocampal circuitry relies on newly-formed dendritic branches of pre-matured neurons that reach the dentate gyrus (DG) molecular layer (ML), where they receive inputs from the entorhinal cortex (EC) lateral (LPP) and medial (MPP) perforant path, forming novel synaptic connections [4]. These cells are able to project their axons to the *Cornu Ammonis* 3 (CA3), further establishing the hippocampal circuitry. Besides this tight communication with EC fibers, these newly-generated neurons also receive inputs from DG mature neurons [3]. Although physiological formation of newly-generated neurons tends to decrease with age in a slow pace, addition of a small number of these cells is critical for DG-dependent memory processing due to their enhanced excitability [3]. Adult neurogenesis, in particular the maturation of newly-generated neurons, is regulated by the action of epigenetic mechanisms, including DNA methylation, histone modification and transcriptional feedback loop (reviewed in [5]). Wnt/β-catenin signalling is another major player in neurogenesis [6, 7]. In this respect, Wnt/β-catenin signalling was previously shown to be involved in the maturation of the newly-generated dendritic tree [7, 8]. In turn, epigenetic mechanisms and Wnt/β-catenin pathway are influenced by environmental factors, including dietary exposures and nutritional status [9, 10]. Discovering dietary tools that promote neurogenesis, namely the efficient integration of newly-generated neurons, may prove to be essential for the maintenance of the brain cognitive reserve and provide the brain with means to cope with several insults.

*Coriolus versicolor* (CV) is a common healthful mushroom that has been used for thousands of years in traditional oriental therapies [11]. Recent studies suggested that this mushroom is endowed with antitumoral, anti-inflammatory, antioxidant, antibacterial and immunomodulatory properties [12–14]. CV can be presented in the form of biomass or extract. Unlike the extract form, originated from concentrated extracts of the fruiting bodies, the biomass comprises mycelia and primordia, which renders it more resistant to proteolytic enzymes of the digestive tract [15]. Due to its enrichment in enzymes with immune-enhancing activity and other substances including β-glucans, which are likely to act in a synergistic manner, CV biomass is seemingly more effective in promoting the detoxification of reactive oxygen species (ROS), thus preventing oxidative stress, when compared with CV extracts [16]. Recently, CV biomass administration was shown to promote an up-regulation of lipoxin A4 (an anti-inflammatory mediator) and an increase in the levels of redox-sensitive proteins involved in the cellular stress response, such as Hsp72, heme oxygenase-1 and thioredoxin, in several brain areas, namely the cortex and the hippocampus [17]. Moreover, CV polysaccharide administration was shown to improve spatial memory in a mouse model of Alzheimer's disease (AD), by increasing the antioxidant activity, through an increase in superoxide dismutase (SOD) and catalase (CAT) mRNA levels, and inhibition of pro-inflammatory cytokines, namely interleukin-1β (IL-1β), interleukin-6 (IL-6) and tumor necrosis factor-alpha (TNF-α) [18].

Although a substantial number of reports highlight the beneficial potential of CV administration, much has yet to be performed to uncover its positive effects on hippocampal neurogenic reserve, as well as cellular and molecular mechanisms involved in these processes. Therefore, the present study was conducted to evaluate whether CV biomass oral administration promotes hippocampal neurogenic reserve under normal/physiological conditions. To achieve this purpose, we quantified hippocampal DG and subgranular zone (SGZ) volume and the number of hippocampal newly-generated neurons, and analyzed the differentiation features of these immature neurons in CV-administered wild-type mice.

Overall, our data strongly suggest that CV dietary supplementation promotes hippocampal neurogenic reserve through an increase in dendritic arborization of newly-generated neurons, which is accompanied by an increment in cytoplasmatic and nuclear β-catenin levels. This increase in dendritic complexity may translate into enhanced cognitive reserve.

## RESULTS

To evaluate whether *Coriolus versicolor* (CV) biomass promotes hippocampal neurogenic reserve, 2.5 month-old mice were administered with 200 mg/kg every day for 2.5 months. During CV administration, animals were monitored in terms of body weight and food and water consumption (Table 1). No significant differences were registered either on the percentage of weight gain, or on total body weight between the two experimental conditions. Indeed, food consumption remained equivalent between controls and CV-administered mice, but the latter mice consumed less water than the controls.

**Table 1:** Effects of daily CV administration (200 mg/kg body weight) on animals' body weight and food and water consumption

	Saline	CV
Final body weight (g)	27.6 ± 0.4	27.0 ± 0.4
Weight gain (%)	8.9 ± 2.0	9.3 ± 1.5
Food intake (g)	3.2 ± 0.1	3.1 ± 0.1
Water intake (mL)	5.0 ± 0.2	4.1 ± 0.2^***^

a:data are expressed as mean SEM for 10 animals per group. Mann–Whitney test: ^***^p < 0.001 vs saline.

b:CV: *Coriolus versicolor.*

As a measure of the effect of CV administration on the hippocampal neurogenic reserve, we quantified the volume of granular cell layer (GCL) and subgranular zone (SGZ) of the hippocampal dentate gyrus (DG), proliferation, number of hippocampal newly-generated neurons and analyzed the differentiation features of these immature neurons from saline- and CV-administered mice. Levels of β-catenin were also measured in DG newly-generated immature neurons.

### GCL and SGZ volumes, proliferation and number of newly-generated neurons remain unchanged after CV administration

**Figure 1:**
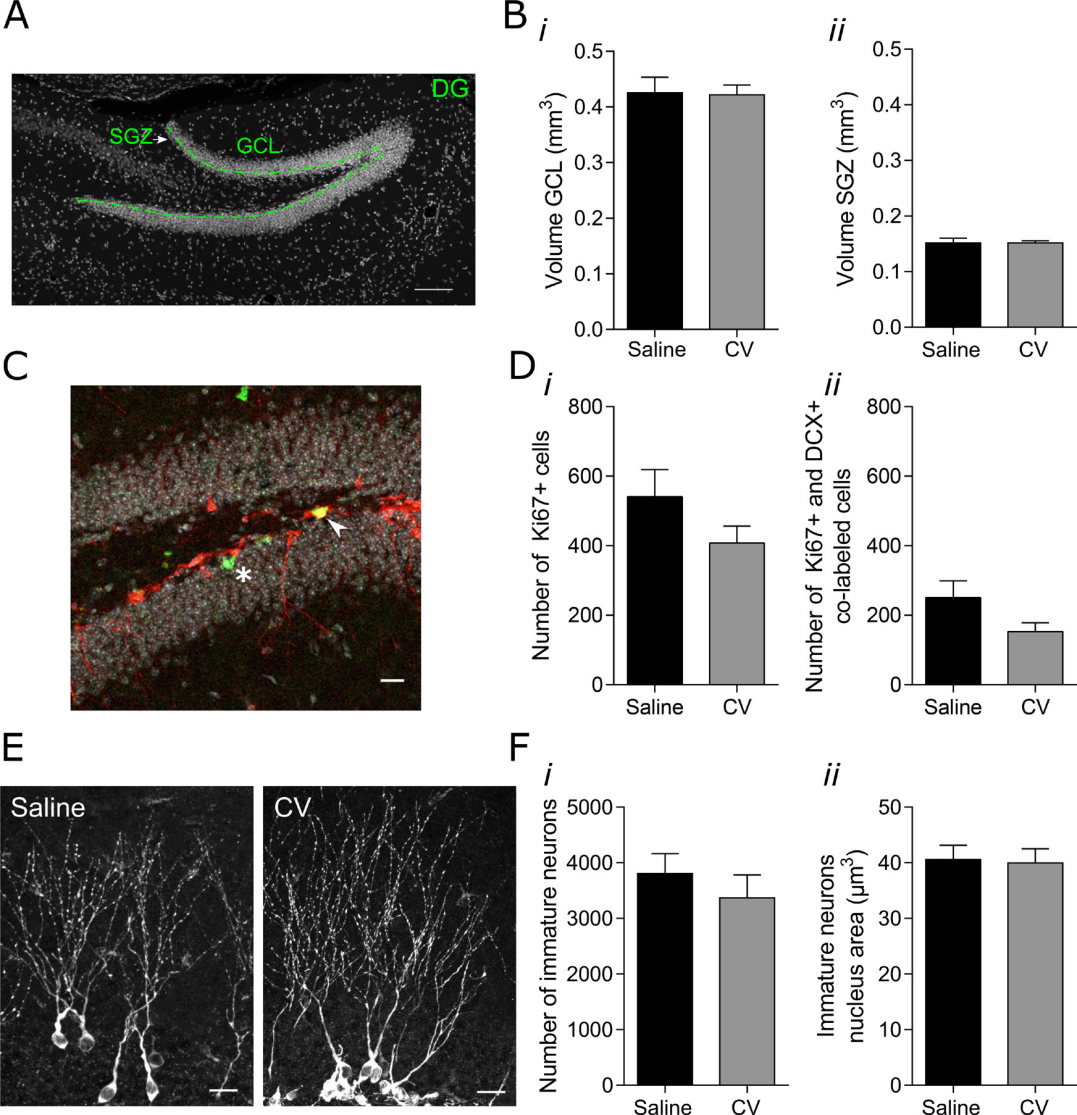
Effect of CV administration on the volumes of the GCL and SGZ of the hippocampal DG, on proliferation of DCX-positive cells and on the number and nucleus area of immature neurons in the DG of mouse hippocampus (**A**) Confocal microscope image showing the SGZ and GCL of the hippocampal DG. Cell nuclei were stained with Hoechst 33342 (in grey). Green line represents the separation between the SGZ and GCL. Scale bar: 50 μm. (**B**) Volumes of GCL (*i*) and SGZ (*ii*) (unpaired *t* test or Mann Whitney test). (**C**) Confocal microscope image showing Ki67- and DCX-positive cells. The arrow indicates a cell expressing both Ki67, a proliferation marker (in green) and DCX (in red), demonstrating a proliferating neuroblast. The asterisk indicates a cell expressing Ki67 and negative for DCX, corresponding to a different type of cell in proliferation. Scale bar: 20 μm. (**D**) Quantification of the number of Ki67-positive cells (*i*) and Ki67-positive cells co-labeled with DCX-positive cells in the GCL of the hippocampal DG (*ii*) (Mann–Whitney test). (**E**) Confocal microscope images showing immature neurons in the hippocampal DG. Immature neurons were stained with DCX antibody (in grey). Scale bar: 20 μm. (**F**) Quantification of the number of immature neurons in the GCL (*i*) and immature neurons nucleus area (*ii*) (unpaired *t*-test). Data are expressed as mean ± SEM. of 9-10 mice per group for GCL and SGZ volumes, 5 mice for the number of Ki67-positive cells and Ki67-positive and DCX-positive co-labeled cells, or 5-6 mice for the number of immature neurons and immature neurons nucleus area.

First, we analyzed the effect of 2.5 months of CV administration in the volume of GCL and SGZ layers of the hippocampal DG (Figure 1A). No differences in the volume of the GCL (Figure 1B, *i*) and SGZ (Figure 1B, *ii*) were observed between controls and CV-administered 5 month-old mice. Proliferation was further studied by counting the number of cell expressing Ki67, a known marker of proliferation that is expressed in all phases of the active cell cycle of proliferating cells (G1, S, G2 and M) [19] (Figure 1C and 1D). We specifically analyzed the proliferation associated to the late stage of neurogenesis. Therefore, cells co-expressing Ki67 and doublecortin [DCX; a microtubule-associated protein expressed in early neuroblasts and new neurons and involved in neuronal migration and differentiation [20–22]] were counted (Figure 1C and 1D). Total cell number was mathematically estimated relatively to the entire hemisphere (as described in ‘Materials and Methods' section). Since immature neurons correspond to the postmitotic phase of DCX-positive cells, the majority of DCX & Ki67-positive cells can be considered as proliferating neuroblasts [23]. No differences were observed neither in the general proliferation (Ki67-positive cells; Figure 1D, *i*) nor in the proliferation of neuroblasts (Ki67- and DCX-positive cells, Figure 1D, *ii*) between control and CV-supplemented mice. Moreover, and concordantly with proliferation data, both the number of GCL immature neurons (Figure 1E and 1F, i), and the nucleus area of these cells (Figure 1E and Figure 1F, *ii*) were similar between controls and CV-administered mice.

### CV administration promotes dendritic branching of immature neurons

**Figure 2:**
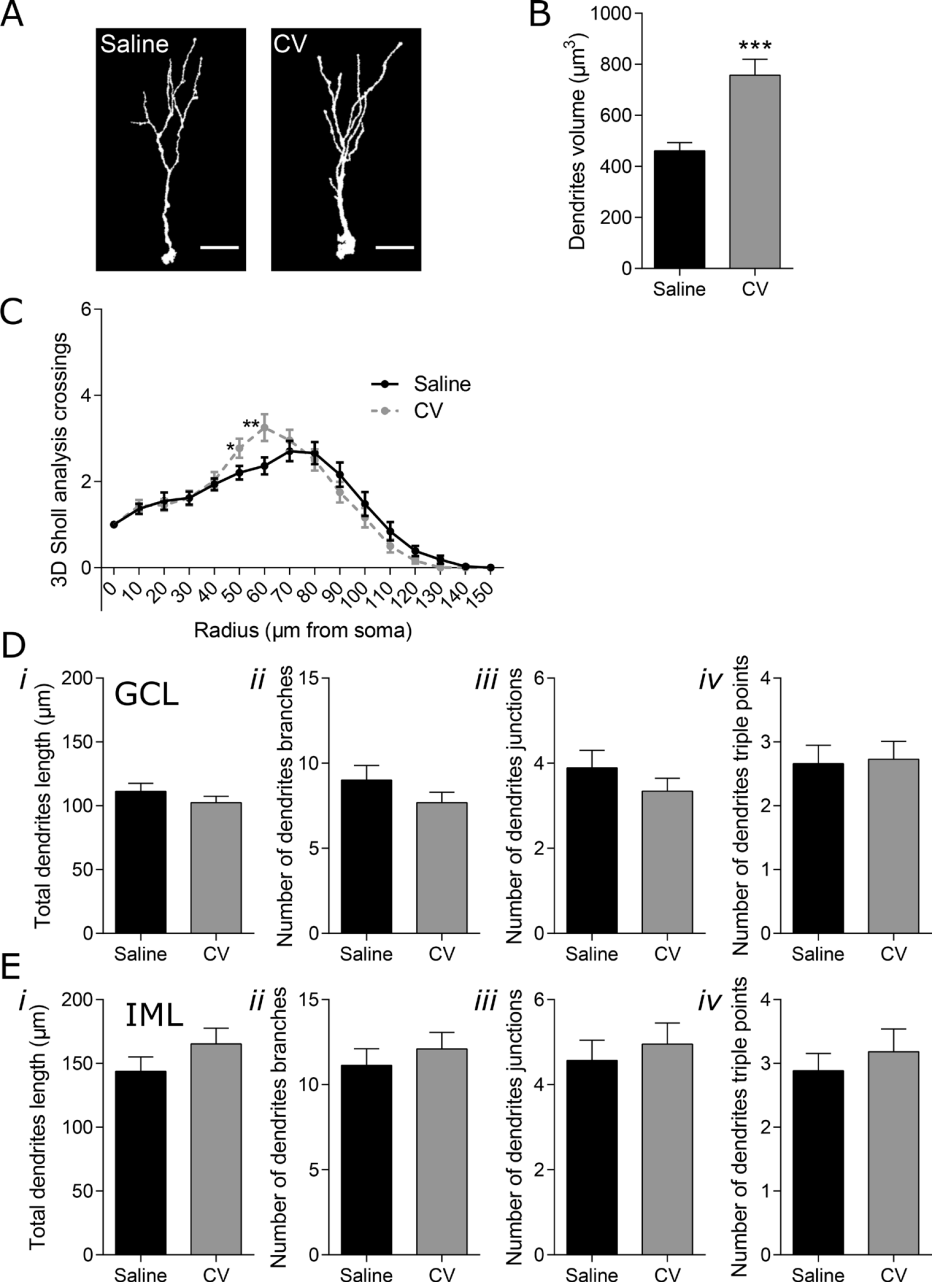
Effect of CV on the morphology of DG short immature neurons of mouse hippocampus (**A**) Three dimensional (3D) reconstructions of short immature neurons (EF DCX-positive cells that do not reach the O/MML). Scale bar: 20 μm. (**B**) Dendrites volume (Mann–Whitney test: ^***^p < 0.001 vs saline). (**C**) 3D Sholl analysis (one-way ANOVA with repeated measures and Bonferroni *post hoc* test: ^*^p < 0.05; ^**^p < 0.01 vs saline). Dendrites length (*i*), number of neuronal branches (*ii*), number of neuronal junctions (*iii*) and number of triple points (*iv*) in the GCL (**D**) or in the IML (**E**) of the hippocampal DG. Data are expressed as mean ± SEM of 44 cells per group.

To assess dendritic complexity, immature neurons were stained for DCX. A requirement for the successful synaptic integration and subsequent functionality of newly-generated neurons is the sprouting and extension of dendrites into the ML of the dentate gyrus (DG), where the synaptic connections are formed [24]. Thus, measurement of dendritic complexity is an indicator of neuronal differentiation in hippocampal adult neurogenesis. To analyze the complexity of newly-generated neurons, DCX cells with morphology of immature neurons (EF-DCX cells) were 3D reconstructed and analyzed by Sholl analysis [25]. Both the maturation stage and the localization of dendrites and establishment of connection in the specific layers of the ML, namely inner molecular layer (IML) and outer and medial molecular layer (O/MML), leads to distinct types of response by the immature neurons (for review [26, 27]). Therefore, EF-DCX cells with dendrites reaching or not the DG O/MML were identified, herein named as “long” or “short” immature neurons, respectively. Sholl analysis of short immature neurons revealed a positive effect of CV administration in the number of dendritic intersections, in the radius 50 and 60 μm, reflecting a slight increase in the dendritic complexity of these cells (Figure 2C). In terms of other features of complexity, CV did not alter the total dendritic length, number of dendritic branches, branch junctions or the number of junctions with three branches (dendrites triple points) in both the GCL and IML of short cells, when compared to control mice (Figure 2D, *i–iv* and 2E, *i–iv*). Importantly, the positive alterations found in the Sholl analysis of short immature neurons from CV-administered mice were accompanied by a significant increase in the neuronal volume of short immature neurons (~1.6 fold), when compared to control mice (Figure 2A and 2B).

**Figure 3:**
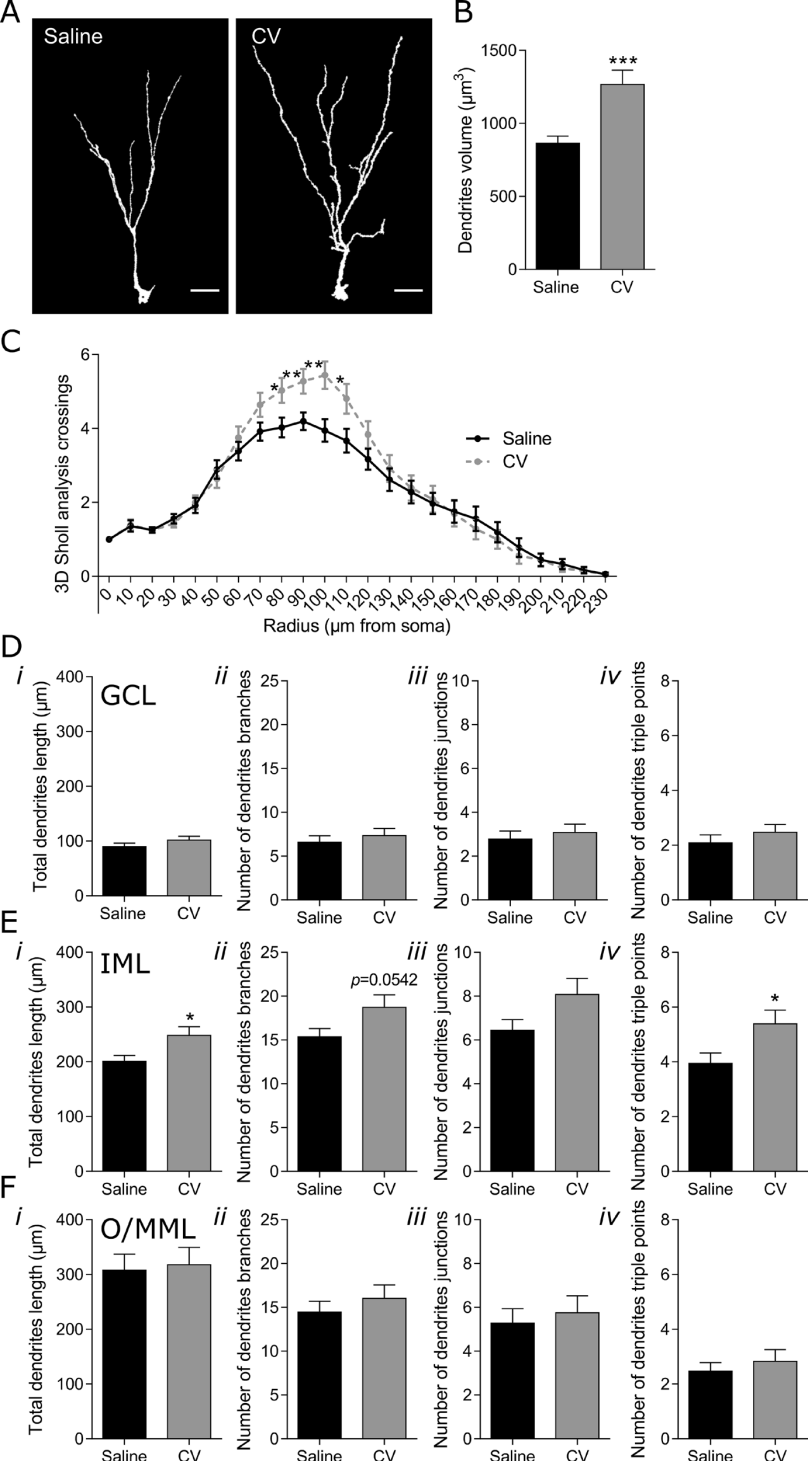
Effect of CV on the morphology of DG long immature neurons of the mouse hippocampus (**A**) Three dimensional (3D) reconstructions of long immature neurons (EF DCX-positive cells that reach the O/MML. Scale bar: 20 μm. (**B**) Dendrites volume (unpaired *t* test: ^***^p < 0.001 vs saline). (**C**) 3D Sholl analysis (one-way ANOVA with repeated measures and Bonferroni *post hoc* test: ^*^p < 0.05, ^**^p < 0.01 vs saline). Dendrites length (*i*), number of neuronal branches (*ii*), number of neuronal junctions (*iii*) and number of triple points (*iv*) in the GCL (**D**), IML (**E**) and O/MML (**F**) of the hippocampal DG (unpaired *t* test or Mann–Whitney test: ^*^p < 0.05 *vs* saline). Data are expressed as mean ± SEM of 36 cells per group.

In the case of immature neurons reaching the O/MML, a more pronounced effect of CV administration was found in the Sholl analysis of the dendritic arborization, namely in radiuses 80 to 110 μm (Figure 3C). The significant increase in the number of intersections in these radiuses strongly suggests a specific effect on the dendrites located at the DG IML. This was further confirmed by the observation that CV administration strongly contributed to the significant increase in the length and number of junctions with three branches (dendrites triple points) of dendrites located at the IML (Figure 3E, *i* and *ii*). In addition, an almost statistical increase in the number of branches (*p* = 0.0542) was found in the DG IML of CV-administered mice (Figure 3E, *iii*). The fact that no alterations were found in total dendritic length, number of dendritic branches, branch junctions and triple points, in both the GCL and O/MML (Figure 3D, *i-iv* and 3F, *i-iv*), further shows the specific effect of CV administration on the long immature neurons dendrites located at the IML. Finally, a significant increase in neuronal volume (~1.5 fold) was found in long immature neurons from CV-administered mice, when compared to control mice (Figure 3A and 3B).

### Diet supplementation with CV upregulates β-catenin levels both in the nucleus and cytoplasm of DG immature neurons

**Figure 4:**
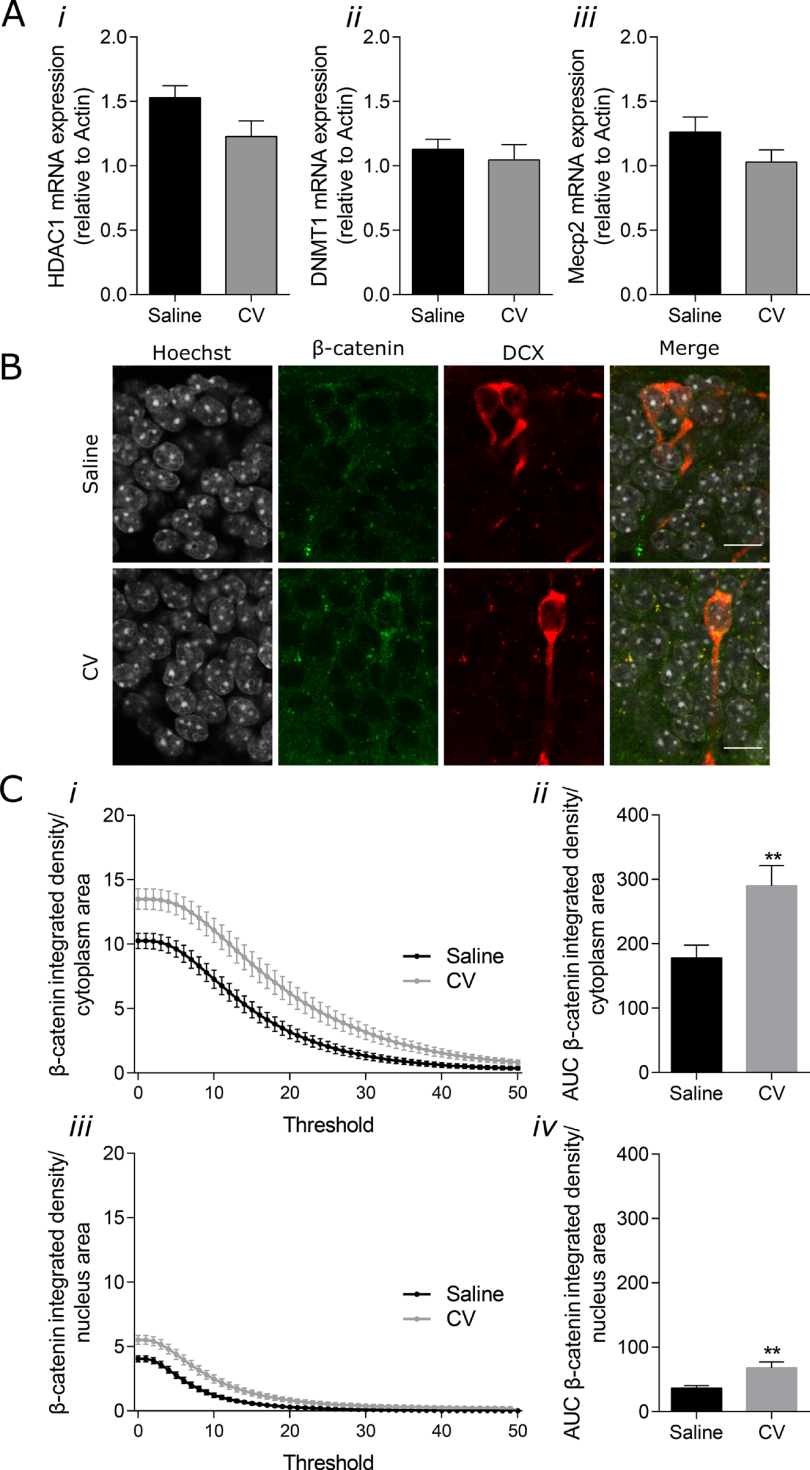
Effect of CV on mRNA expression of HDAC1, DNMT1 and Mecp2 in the mouse hippocampus and on β-catenin levels in the cytoplasm and nucleus of DG immature neurons (**A**) mRNA expression levels of HDAC1 (*i*), DNMT1 (*ii*) and Mecp2 (*iii*) in the mouse hippocampus. (**B**) Confocal microscope images showing β-catenin staining in immature neurons in the hippocampal DG (nuclei in grey, DCX in red and β-catenin in green). Merge images show the presence of β-catenin in the nucleus and cytoplasm of immature neuron. Scale bar 20 μm. (**C**) β-catenin integrated density *per* area (from threshold 1-50) (*i* and *iii*) and area under the curve (AUC) (*ii* and *iv*) in the cytoplasm (*i* and *ii*) and nucleus (*iii* and *iv*) of immature neurons from the DG (Mann–Whitney test: ^**^p < 0.01 vs saline). Data are expressed as mean ± SEM of 10 mice *per* group for mRNA expression, and 43 cells from 6 mice *per* group for β-catenin levels quantification.

Among the different molecules found to intervene in the epigenetic regulation of adult neurogenesis, three types of regulators have been identified to interfere with the maturation and integration of newborn neurons: DNA (cytosine-5)-methyltransferases (DNMTs), Methyl-CpG binding protein 2 (MeCP2) and histone deacetylases (HDACs) (reviewed in [5]). In line with this reasoning, we evaluated the mRNA levels of DNMT1, HDAC1 and MeCP2 in the hippocampal samples of control and CV-supplemented mice. As depicted in Figure 4A, no significant differences were found on mRNA expression of these genes. Since the number of immature neurons accounts for a very small portion of the entire hippocampus, it is highly possible that the specific effect of CV administration on these epigenetic regulators from immature neurons is masked in these samples.

On the other hand, it was shown that activation of Wnt signaling in the SGZ increased adult neurogenesis, whereas its inhibition caused a reduction in proliferation and neuronal differentiation [6]. Therefore, we quantified the β-catenin levels in the nucleus and cytoplasm of the newly-generated neurons showing increased arborization, by immunohistochemistry. A clear increase in β-catenin levels was observed in both the cytoplasm (Figure 4C, *i* and 4C, *ii*) and nucleus (Figure 4C, *iii* and *iv*) of the immature neurons from CV-administered mice, when compared to control mice (Figure 4B and 4C), suggesting that CV may stimulate this signalling pathway, which may in turn be responsible for the increase in maturation of newly-generated neurons, promoted by CV supplementation.

## DISCUSSION

The brain is constantly challenged by different insults, such as disease states, stress, injury and/or aging. In recent years, it has been hypothesized that the capacity of the brain to cope with these insults, and maintain normal function, is highly dependent on the brain cognitive reserve [28, 29]. This capacity of the brain to develop compensatory mechanisms is positively or negatively modulated by exogenous factors, such as lifestyle, exercise or nutritional factors, which can determine healthy aging or the development of age-dependent neurodegenerative diseases [30, 31]. Many authors have pointed out the relevance of cognitive reserve and lifestyle for diseases such as AD, Parkinson's disease (PD) and Huntington's disease (HD) [28, 32–34]. Brain cognitive reserve is a theoretical concept that may be indirectly measured [35]. Some brain properties directly related to brain plasticity have been suggested to be constituents of the brain cognitive reserve and, thus, indicators of its state, such as volume of brain structures, neuronal number, neuronal complexity and connectivity [28]. Importantly, hippocampal adult neurogenesis has been described to contribute to the volume of the DG, increase and maintain the number of granule cells [36], and mainly the dendritic tree of adult newly-generated neurons adapts to behavioural demands in the DG [37]. Therefore, as previously proposed, hippocampal adult neurogenesis may be considered as part of the brain cognitive reserve and reflects its state [38]. Numerous factors can shape the neurogenic process and identifying such factors, which positively modulate the production and integration of new neurons, may prove to be essential for the response of the brain to insults that may lead to neuropathology. In this study, it was demonstrated that oral supplementation with CV biomass promotes an increase in the arborization of the dendritic tree of hippocampal immature neurons, which is more evident in newly-generated neurons with dendrites reaching the DG O/MML. Data revealed a modest increase in the number of dendrite intersections in the Sholl analysis of immature neurons with dendrites ending in the IML from CV-administered mice, when compared to control mice. These cells may correspond to an early stage of differentiation that, with time, might give rise to cells with a similar morphology to the ones observed in immature neurons reaching the O/MML (long immature neurons). During the first week of cell development, DG newly-generated neurons extend their dendritic processes into the IML, not reaching the OML [39], with a morphology very similar to that of cells called short immature neurons. During the following week, these cells extend their dendritic branching and reach the O/MML [40]. After this period, and in the next two weeks, these cells may develop a complex dendritic arbor, with the development of spines and the first glutamatergic inputs, reaching the maximum level of maturity [40]. This level of differentiation will then allow the establishment of contacts with the existing entorhinal cortex (EC) [39]. Both Sholl analysis and complexity parameters indicate that CV supplementation induces an increase in the complexity of long immature neurons dendrites residing in the IML, including their length. Neurons in the DG ML can receive inputs from different afferent sources. In the case of O/MML neurons they establish contacts with fibers from the lateral or medial EC (innervating the OML, respectively) [27]. Dendrites located at the IML in turn receive inputs mainly from the mossy cell axons [27]. A possible role for mossy cells are their contribution for pattern separation, a process involving the crossing of information coming from the EC [27]. Since the role of mossy cells on hippocampal neurogenesis and its contribution for functionality is very limited, we can hypothesize that immature neurons from CV-administered mice, with more dendrites at the IML, may establish contacts with these circuits and potentiate the pattern separation process.

Although we did not observe any alteration in GCL and SGZ volume, as well as in proliferation of neuroblasts and number of immature neurons, parameters used to measure hippocampal neurogenic reserve, it is possible to infer that a more prolonged treatment and/or an increase in the dose of CV supplementation would result in an increase of both number and volume of neuronal cells. In view of the fact that the available literature is very sparse or inexistent regarding the effect of CV administration on proliferation and neuronal survival, additional experiments addressing this issue are warranted. Nonetheless, given the potential role of adult hippocampal neurogenesis, especially the integration of new neurons, for pattern separation and spatial learning [38, 41], an extended dendritic arborization of newly-generated neurons is expected to enhance the probability of integration into the local hippocampal circuitry. Therefore, the increased dendritic complexity seen herein is suggestive that oral CV administration has a positive effect in the hippocampal neurogenic reserve, which may prevent cognitive impairment upon deleterious events that accompany aging and neurodegenerative diseases. However, future studies will be necessary to address these issues. Interestingly, the positive effect exerted by CV administration on dendritic complexity is similar to that obtained with environmental enrichment (EE) [42, 43]. In fact, EE has been widely described to promote hippocampal neurogenesis and to enhance cognition in models of disease, such as AD [44].

Despite the potential health effects of CV, its molecular targets are still poorly known. Numerous signals regulate hippocampal neurogenesis in its distinct stages, both by intrinsic factors, namely intracellular signalling pathways, microenvironment or cell to cell interaction; and extrinsic factors, such as diet (for review [26]). One of the signalling pathway that plays an important role in the differentiation of neuroblasts into mature neurons is the Wnt/β-catenin signalling pathway [7]. In the canonical Wnt/β-catenin pathway, β-catenin cytoplasmic levels are dynamically regulated by a complex composed of adenomatous polyposis coli (APC), Axin, glycogen synthase kinase-3β (GSK-3β) and casein kinase 1 (CK1) that phosphorylates β-catenin, leading to its ubiquitination and proteasomal degradation [45, 46]. When Wnt is present and interacts with Frizzled (Fzd) family receptors, this complex is inhibited, allowing the increase in cytoplasmic β-catenin levels and its translocation to the nucleus [45]. In the nucleus, β-catenin interacts with T cell factor/lymphoid enhancer factor-1 (TCF/Lef1) and activates the TCF/Lef1 transcription complex, promoting cellular differentiation [45, 47]. Interestingly, CV supplementation leads to an increase of both cytoplasmic and nuclear levels of β-catenin in immature neurons from hippocampal DG, suggesting that this protein may be a key molecule responsible for the increase in dendritic complexity in these cells. To our knowledge, this is the first time that CV supplementation has been described to alter the Wnt/β-catenin signalling pathway, by increasing β-catenin levels. On the other hand, although epigenetic mechanisms are amenable to modulation by diet [9], CV supplementation does not seem to change the hippocampal expression of genes that are epigenetic regulators of adult neurogenesis. However, since the number of immature neurons accounts for a very small portion of the entire hippocampus, it is highly possible that any specific effect of CV administration on these epigenetic regulators in immature neurons might be masked in these samples representing total hippocampus. In pathological situations, it is possible that CV also exerts a protective effect on hippocampal adult neurogenesis through its action as an antioxidant. In fact, it was suggested that an acetone CV extract was endowed with antioxidant properties [48]. These authors further demonstrated that this extract was enriched in furfural, furfuryl alcohol, 2-methoxy-4-vinylphenol and 2,6-dimetoxy-4-vinylphenol. Furfural and furfuryl alcohol are non-phenolic compounds whereas 2-methoxy-4-vinylphenol and 2,6-dimetoxy-4-vinylphenol are phenolic compounds. Additionally, CV contains immunonutrients proteoglycans (eg. polysaccharopeptide Krestin - PSK), which have antioxidant properties [16]. Although the impact of these compounds was never tested in neurogenesis, we may argue that the positive effect of CV seen herein may be the result of the synergistic action of β-glucans, proteoglycans, furfurals and the phenolic compounds 2-methoxy-4-vinylphenol and 2,6-dimetoxy-4-vinylphenol. Moreover, as previously mentioned, CV administration can upregulate lipoxin A4, which in turn promotes an increase in redox-sensitive neuroprotective proteins involved in cellular stress response, such as Hsp72, heme oxygenase-1 and thioredoxin [17]. Although slight production of ROS may occur along the neurogenic process and is therefore physiological [49], prolonged oxidative stress, which typically occurs in aging and neurodegenerative diseases, may contribute for neurogenesis impairment [50].

In conclusion, our data demonstrate that supplementation with CV biomass promotes the increase in the levels of β-catenin in the nucleus and cytoplasm of hippocampal newly-generated neurons. It is tempting to argue that this may contribute to the increase in dendritic arborization, and, therefore, to the reinforcement of the neurogenic cognitive reserve. This improvement may thus promote the brain capacity to cope with insults, favouring normal brain function.

## MATERIALS AND METHODS

### Animals and ethics statement

Wild-type mice (C57BL/6 × 129 background) were bred and maintained at CNC-Faculty of Medicine animal house (license n° 520.000.000.2006, from the Portuguese animal welfare authorities). Two and half month-old male mice were randomly divided in 2 experimental groups (*n* = 10 per group): i) Saline-administered animals and ii) CV-administered animals (200 mg/kg body weight, suspended in saline (as in [11]). Mice were grouped in cages of 4–6 animals and kept in the same room, under standard laboratory conditions (21 ± 2°C, 12 h light/dark cycle starting at 07:00 am, *ad libitum* access to food (# 4RF21A, Mucedola, Milanese, Italy) and water. CV biomass (supplied by Mycology Research Laboratories Ltd, Luton, UK) was micronized to allow its administration by oral gavage with flexible cannulas (22 Gauge, 25 mm length; B. Braun, Bethlehem, USA). Saline vehicle was administered to control mice. The administration of CV or saline was performed every day for 2.5 months. During this period, liquid consumption was monitored every two days, while food consumption and animal weight were monitored once a week. All procedures were performed to minimize exposure to stress and suffering, in accordance with the approved animal welfare guidelines of the institutional animal house (local welfare approval: ORBEA_140_2016/15072016) and European legislation (European directive 2010/63/EU).

### Preparation of *Coriolus versicolor* biomass low size particles

Before size reduction procedures, the original CV powder was analyzed by Attenuated Total Reflectance Infrared Spectroscopy (ATR-FTIR) and its particle size distribution was determined by laser diffraction (data not shown). Then, the powder was submitted to manual pulverization with a mortar, in order to reduce particle size. After pulverization, sieving was performed with a Retsch AS 200 sieve shaker. With this procedure four different granulometry (particle size ranges) were obtained: 1) >355 μm; 2) 355≤ particle size ≤125 μm; 3) 125 < particle size ≤90 μm and 4) <90 μm. After sieving, a sample of each granulometry was analyzed by ATR-FTIR and laser diffraction to determine the particle size distribution and the results were compared with the original powder. ATR-FTIR results showed that all of them shared the same structural composition of the original powder, and the laser diffraction analysis confirmed the reduction in mean and median particle size that was achieved with this procedure (data not shown). By using the fraction with the lowest granulometry (<90 μm), it was possible to prepare a suspension that did not clog the oral gavage cannulas used to administer the biomass to the animals.

### Immunofluorescence staining

Mice were deeply anesthetized with sodium pentobarbital (70 mg/kg i.p.) and perfused intracardially with 0.9% NaCl, for 4 min. Brain were immediately dissected and the left hemisphere postfixed with 4% paraformaldehyde in phosphate buffered saline (PBS; containing in mM: 137 NaCl, 2.7 KCl, 1.8 KH_2_PO_4_, 10 Na_2_HPO_4_.2H_2_O, pH 7.4), for 24 h at 4°C. Hemispheres were rinsed twice with PBS and cryoprotected with 30% w/v sucrose in PBS, at 4°C, until complete submersion. Cryoprotected hemispheres were snap frozen, kept at –80°C, until sectioning. Coronal slices with 40 μm were obtained using a cryostat-microtome (CM3050S; Leica, Mannheim, Germany) and collected in six series. Slices were then stored at –20°C in anti-freeze solution [30% glycerol (v/v) and 30% polyethylene glycol (v/v) in 0.1 M phosphate buffer, pH 7.4].

Serial slices were used and rinsed overnight (O.N.) at 4°C and then 3 × 10 min at room temperature (RT) with PBS. Slices were blocked with blocking solution [3% bovine serum albumin (BSA, w/v) and 1% Triton X-100 in PBS], for 1 h at RT. Sections were then incubated with primary antibodies [for triple DCX/postsynaptic density protein 95 (PSD95)/Ki67 staining: goat anti-DCX (1:500; Clone C-18, Sc-8066, Santa Cruz Biotechnologies), mouse anti-PSD95 (1:2,000; clone K28/43, #MABN68, Merck Millipore) and rabbit anti-Ki67 (1:2,000; ab16667, Abcam); for double DCX/β-catenin staining: goat anti-DCX (1:500; Clone C-18, Sc-8066, Santa Cruz Biotechnologies) and goat anti-β-catenin (1: 500; ab32572, Abcam)] prepared in blocking solution for 72 h, at 4°C. Sections were rinsed 3 × 10 min with PBS and incubated for 2 h at RT with the appropriate secondary antibodies [Alexa Fluor^®^ 568 donkey anti-goat IgG (# A-21082; Thermo Fisher Scientific), Alexa Fluor^®^ 488 donkey anti-mouse IgG (# A-21202; Thermo Fisher Scientific), Alexa Fluor^®^ 647 donkey anti-rabbit IgG (#A-31573; Thermo Fisher Scientific) and Alexa Fluor^®^ 488 donkey anti-rabbit IgG (#A-21206) at a dilution of 1:1000] prepared in PBS supplemented with Hoechst 33342 (0.2 μg/ml; # H1399, Thermofisher Scientific, Waltham, MA USA). Sections were finally rinsed 3 × 10 min with PBS and mounted with anti-fading medium (Fluoroshield Mounting Medium, # ab104135, Abcam).

### Volume and cell number quantifications

GCL and SGZ areas (from bregma –4.04 mm to bregma –0.94 mm) [51] were measured using confocal tile images of the Hoechst 33342 staining in the DG, obtained with an inverted Zeiss LSM 710 confocal microscope, equipped with a Plan-Apochromat 20×/0.8 NA objective and a 0.7 × digital zoom. Consecutive slices distanced 480 μm were used for this evaluation. SGZ and GCL areas were measured using Fiji software (https://fiji.sc/Fiji) [52]. A line was drawn in the inner limit of the GCL and each side of the line was thickened 12.5 μm to define the SGZ. The estimation of the entire GCL and SGZ volumes in the left hemisphere were obtained by calculating the sum of the area of all the consecutive pair of measured sections. To this end, section areas were ordered from rostral to caudal and volumes were calculated applying the mathematical formula V = π × h × (R^2^ + r^2^ + R × r)/3 (V for volume, h for the distance between the two slices, r for the radius of the first slice (slice area) and R for the radius of the second slice). The volumes of the rostral end of the GCL and SGZ were obtained by estimating the radius of the most rostral section assuming the linear distribution of the area of the last 2 rostral sections.

The total number of immature neurons was estimated by counting EF (post mitotic and almost mature) DCX-positive cell bodies (DCX-cells with branching in the GCL and ML) in the GCL of consecutive slices distanced 480 μm, representing the entire DG (from bregma –4.04 mm to bregma –0.94 mm) [51]. For this purpose, confocal stack and tile images were obtained with an inverted Zeiss LSM 710 confocal microscope, equipped with a Plan-Apochromat 20×/0.8 NA objective and a 0.7 × digital zoom. Total number of immature neurons was estimated by applying the Abercrombie formula [53]. The same method was applied to estimate the total number of Ki67-positive (corresponding to all cells in proliferation in the GCL) and Ki67-positive and DCX-positive co-labelled cells in the GCL of the left hemisphere (corresponding to neuroblasts in proliferation).

### Analysis of dendritic morphology

To analyze the dendritic morphology of immature neurons, confocal stack images were obtained with an inverted Zeiss LSM 710 confocal microscope, equipped with a Plan-ApoChromat 40×/1.4 NA oil-immersion objective and a 0.7 × digital zoom. Three dimensional (3D) reconstruction was performed using the Simple neurite tracer plugin [54] working on the image processing package Fiji [52]. Immature neurons were grouped in two cell types: i) short (EF-DCX cells that do not reach the O/MML; ii) long (EF-DCX cells that reach the O/MML). Cell type identification was based on the identification of dendrites localization in the IML and O/MML, having PSD95 staining as a reference. 3D Sholl analysis ImageJ plugin (https://fiji.sc/Sholl_Analysis) was used to quantify the number of intersections between dendrites and the surface of spheres with a radius increment of 10 μm. Dendrite length and complexity was analyzed with the imageJ plugins Skeletonize3D (https://imagej.net/Skeletonize3D) and AnalyzeSkeleton (https://imagej.net/AnalyzeSkeleton).

### RNA extraction, reverse transcription and quantitative real time PCR

The RNA content of mouse right hippocampi was extracted using Purezol™ (Bio-Rad, Hercules, CA, USA) reagent as described by the manufacturer's, and quantified using Nanodrop spectrophotometer. Reverse transcription was performed on each RNA sample (500 ng) using the iScript cDNA synthesis kit (Nzytech, Lisbon, Portugal), following the manufacturer's instructions. Gene specific primers used for real time PCR reactions were the following: DNA (cytosine-5)-methyltransferase 1 (DNMT1): forward 5′-CTGCTGTGGAGAAACTGGAA-3′, reverse 5′-TGATTTCCGCCTCAATGATA-3′; methyl CpG binding protein 2 (Mecp2): forward 5′-ATATTTGA TCAATCCCCAGGG-3′, reverse 5′-CTT AGGTGGTTTC TGCTCTC-3′; Histone deacetylase 1 (HDAC1): forward 5′-GACCCTGACAAACGCATCTC-3′, reverse 5′-GTTC TTGCGACCACCTTCTC-3′ and β-actin: forward 5′- GTGACGTTGACATCCGTAAAGA-3′, reverse 5′-GCCG GACTCATCGTACTCC-3′. Quantitative RT-PCR was performed with 10 ng of the cDNA, 400 nM of each primer, and iQ SYBR Green Supermix (Bio-Rad, Hercules, CA, USA). PCR cycles were proceeded as follows: Taq activation (95°C, 3 min), denaturation (95°C, 15 s), and annealing/extension (57°C, 45 s) using the Bio-Rad CFX 96 Real-time system, C1000 Thermal cyclerm (Bio-Rad, Hercules, CA, USA). Melting curve was obtained under 0.5°C increments every 5 s, from 65°C to 95°C, with fluorescence recording after each temperature increment to verify the specificity of the amplification. The relative mRNA levels were estimated using the Bio-Rad CFX manager 2.1 software using β-actin as a reference gene.

### Quantification of β-catenin levels

Single-plane confocal images were obtained using an inverted Zeiss LSM 710 confocal microscope, equipped with a Plan-ApoChromat 40×/1.4 NA oil-immersion objective to quantify β-catenin levels (33 images (*n* = 1–2 cells/image for a total of 43 cells from 6 mice), 34 images (*n* = 1–2 cells/images for a total of 43 cells from 6 mice) for saline and CV, respectively; (from –3.52 mm to bregma –1.34 mm [13]). Fiji software [14] was used to analyze confocal images and an ImageJ macro was created to select the nuclei and soma of DG immature neurons. A second macro was then developed to further select the cytoplasm, by subtracting the region of interest (ROI) corresponding the nuclei from the ROI corresponding to the soma. A final macro was designed to quantify single cell mean integrated density of β-catenin signals, determined at all possible thresholds, and divided by the respective area (published in [55], with some modifications). Results are presented as both the integrate density curves for thresholds 1 to 50 and as the area under the curve (AUC) of these curves (corresponding to the maximum intensity of β-catenin signal).

### Statistical analysis

Data are expressed as means ± standard error of the mean (SEM). Statistical significance was determined using One-Way analysis of variance (One-way ANOVA) with repeated measures ANOVA followed by Bonferroni post hoc test (Scholl Analysis), unpaired student's *t* test for data passing normality tests or non-parametric Mann–Whitney test for data without normal distribution. *p*-values < 0.05 were considered statistically significant. Statistical analysis was performed using GraphPad Prism Software (San Diego, CA, USA) or SPSS for Windows (SPSS Inc., Chicago, IL).

## Abbreviations

3D: three dimensional
AD: Alzheimer's disease
ATR-FTIR: Attenuated Total Reflectance Infrared Spectroscopy
BSA: bovine serum albumin
CA3: *Cornu Ammonis* 3
CAT: catalase
CV: *Coriolus versicolor*
DCX: doublecortin
DG: dentate gyrus
EC: entorhinal cortex
EE: environmental enrichment
GCL: granular cell layer
HD: Huntington's disease
IL-1β: interleukin-1β
IL-6: interleukin-6
IML: inner molecular layer
LPP: lateral perforant path
ML: molecular layer
MPP: medial perforant path
O.N.: overnight
O/MML: outer and medial molecular layer
PBS: phosphate buffered saline
PD: Parkinson's disease
PSD95: postsynaptic density protein 95
ROS: reactive oxygen species
RT: room temperature
SEM: standard error of the mean
SGZ: subgranular zone
SOD: superoxide dismutase
TNF-α: tumor necrosis factor-alpha
